# A Comparative Review of the Cell Biology, Biochemistry, and Genetics of Lactose Synthesis

**DOI:** 10.1007/s10911-021-09490-7

**Published:** 2021-06-14

**Authors:** Anna Sadovnikova, Sergio C. Garcia, Russell C. Hovey

**Affiliations:** 1 Graduate Group in Nutritional Biology, Physician Scientist Training Program, University of California, Davis, CA USA; 2 School of Life and Environmental Sciences, University of Sydney, Sydney, Australia; 3 Department of Animal Science, University of California, Davis, CA USA

**Keywords:** Lactation, Mammary epithelial cells, Alpha-lactalbumin, Lactose synthase complex, Beta-1,4-galactosyl transferase, Oligosaccharide

## Abstract

Lactose is the primary carbohydrate in the milk of most mammals and is unique in that it is only synthesized by epithelial cells in the mammary glands. Lactose is also essential for the development and nutrition of infants. Across species, the concentration of lactose in milk holds a strong positive correlation with overall milk volume. Additionally, there is a range of examples where the onset of lactose synthesis as well as the content of lactose in milk varies between species and throughout a lactation. Despite this diversity, the precursors, genes, proteins and ions that regulate lactose synthesis have not received the depth of study they likely deserve relative to the significance of this simple and abundant molecule. Through this review, our objective is to highlight the requirements for lactose synthesis at the biochemical, cellular and temporal levels through a comparative approach. This overview also serves as the prelude to a companion review describing the dietary, hormonal, molecular, and genetic factors that regulate lactose synthesis.

## Introduction

Milk is essential for mammalian survival, where lactose is one of its major components that is synthesized and secreted by the mammary epithelium, either in its free form or as an oligosaccharide. The concentration of lactose in milk is strongly correlated with the overall volume of milk output. As such, defining the mechanisms that underlie lactose synthesis represents a first step in developing strategies to manipulate and improve the production and composition of milk.

Herein we review the various precursors, genes, proteins, and ions required for optimal lactose synthesis. In doing so, one of our primary objectives is to highlight ways in which various mammals, from marsupials to marine placental therians, ruminants and non-ruminant livestock, rodents, and primates have retained or adapted mechanisms for lactose synthesis to meet the demands of their environment and the needs of their offspring. By reviewing the physiology, biochemistry, and genetics of lactose synthesis through a comparative lens, we also aim to provide a background for the second part of this review where we further explore the extrinsic and intrinsic factors that regulate lactose synthesis.

## The Importance and Variability of Lactose in Milk

**Fig. 1:**
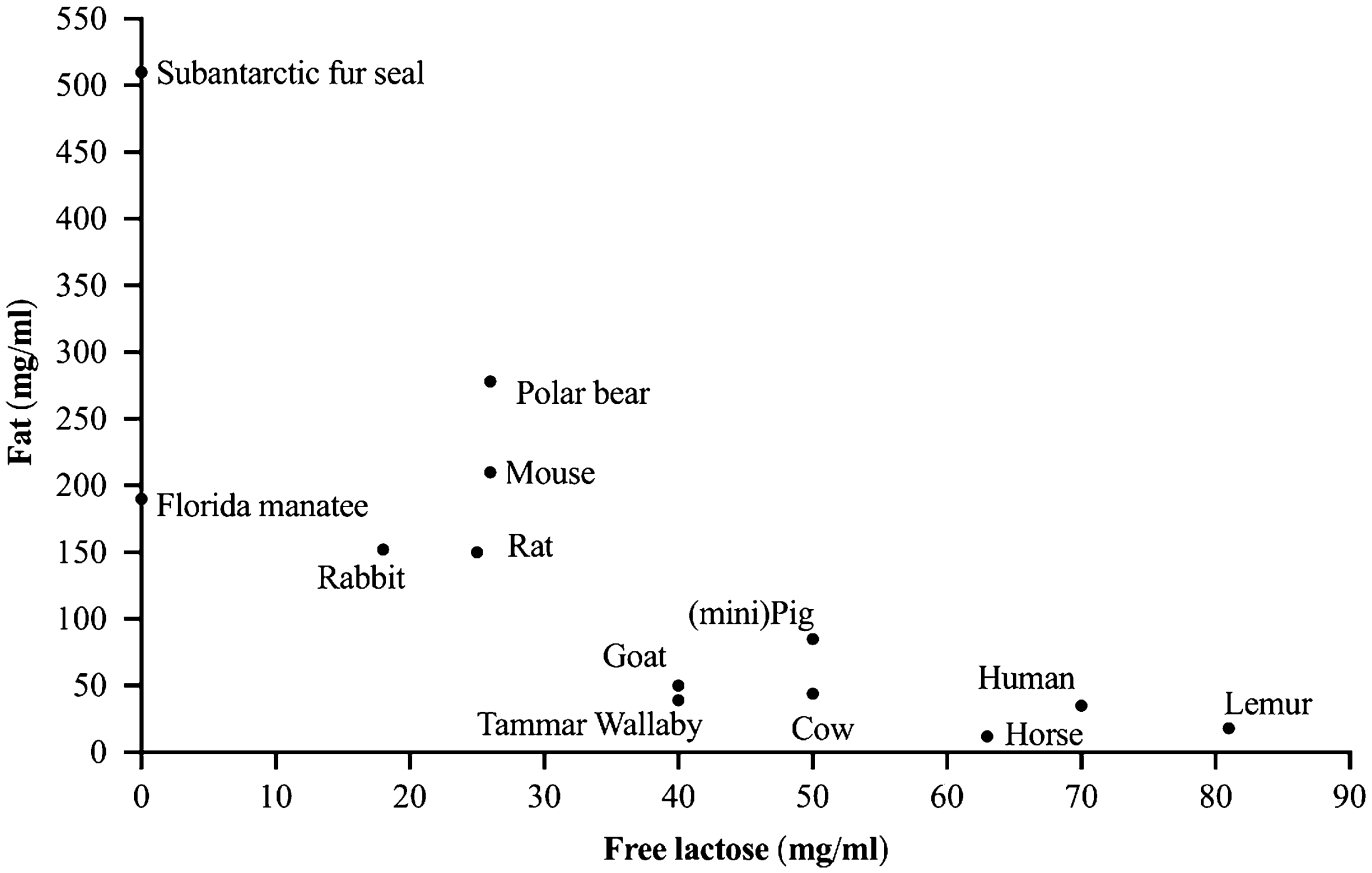
The relationship between lactose and fat content in the mature milk of different species. A more comprehensive graphical presentations of the association between milk lactose and fat concentration have been presented elsewhere [1]. Data for fat and lactose content in the milk of the human, cow, goat, mouse, rat, dog, minipig are presented as the mean of published ranges. Tammar wallaby: Fat (40 mg/ml) was measured at 26 weeks of lactation and lactose (39 mg/ml) between 13 and 34 weeks of lactation [25, 140]; Florida manatee: Fat (190 mg/ml) and lactose (not detected) at 30 weeks and at 2 years of lactation [9]; Human: fat (28–44 mg/ml) and lactose (61–79 mg/ml) between 40 and 180 days postpartum [5]; Lemur: fat (18 mg/ml) and lactose (81 mg/ml) at 72 days postpartum [5]; Cow: fat (33–54 mg/ml) and lactose (44–56 mg/ml) during mid-lactation [6]; Horse: fat (12.1 mg/ml, range 50–200) and lactose (63.7 mg/ml, range 58–70) during mid-lactation [141]; Goat: fat (40 mg/ml) and lactose (32–50 mg/ml) during mid-lactation [6]; Mouse: fat (190–220 mg/ml) and lactose (24–28 mg/ml) in mature milk samples [7]; Rat: fat (140–159 mg/ml) and lactose (11–41 mg/ml) in mature milk samples [7]; Rabbit: fat (152 mg/ml) and lactose (18 mg/ml) in mature milk samples [7]; Dog: fat (24–134 mg/ml) and lactose (29–40 mg/ml) in mature milk samples [7]; (mini)Pig: fat (77–100 mg/ml) and lactose (43–56 mg/ml) in mature milk samples [7]; Subantarctic fur seal: fat (510 mg/ml) and lactose (not detected) in mid-lactation samples [12]; Polar bear: fat (278 mg/ml) and carbohydrate (26 mg/ml) in yearlings mid-lactation sample [142]

One of the most fascinating aspects of milk produced by mammals is the range of its lactose concentration [1]. Free lactose, a disaccharide comprised of glucose and galactose, is the primary carbohydrate in the milk of most placental mammals and comprises more than 80% of all its carbohydrate [2, 3]. Consistently, across nearly all mammalian species, there is a negative relationship between the content of lactose and fat in milk (Fig. 1), which ensures the offspring receives a steady source of calories [1]. For example, human infants receive 40% of their caloric requirements from the approximately 70 g/d of lactose they consume in the first six months of life [4]. In fact, the concentration of lactose in the milk of different primates is consistently high (61 to 89 mg/ml) and accounts for one- to two-thirds of total milk energy [5]. In a similar way, lactose in bovine milk (44 to 56 mg/ml) provides approximately 30% of the calories required by newborn calves [1, 6]. Milk produced by rodents has a lower content of lactose, ranging from 24 to 28 mg/ml in mice, and 11 to 41 mg/ml in rats [7]. Aquatic Pinnipeds, like the otariid species, produce milk devoid of lactose that enables them to avoid involution induced by milk stasis [8], while others like the phocids, vary their production of lactose [9–12]. This broad diversity in the lactose content of milk across species remains an underexplored information treasure trove, not only for better understanding the physiological regulation of lactose, but also for refining an understanding of how nutrients are partitioned across a range of demands and tissues in support of lactation.

Lactose also acts as the primer for oligosaccharide synthesis [13], where it serves as the reducing end of the oligosaccharide core [14]. The > 130 different forms of oligosaccharides in human milk serve as prebiotics for the growth of beneficial bacteria in the infant gut [3, 15]. The abundance of these oligosaccharides and their abundance in milk relative to lactose varies widely across species. For example, the ratio of oligosaccharides to lactose in human milk is between 0.21 and 0.38, where the oligosaccharide content decreases from 23 mg/ml in colostrum to between 5 and 12 mg/ml in mature milk [16]. By contrast, bovine milk contains around 39 oligosaccharides at a concentration from 0.03 to 0.06 mg/ml, which is comparable to that in ovine milk (0.02 to 0.04 mg/ml), albeit lower than that in caprine milk (0.25 to 0.3 mg/ml) [3]. The milk of mice and rats contains a variety of oligosaccharides, where milk from rats, for example, contains more sulfated oligosaccharides [13, 17]. Even though the oligosaccharide content of milk has been characterized for dozens of species [13], the role of these oligosaccharides during neonatal growth and development in non-human mammals remains poorly understood [3, 18, 19].

Oligosaccharides are the primary carbohydrate in the milk of monotremes, marsupials, and in many carnivores, especially among the Arctoidea species (except the domesticated dog) where the ratio of oligosaccharides to free lactose is considerably higher than that found in human or bovine milk. For example, the ratio of oligosaccharides to free lactose ranges from 7:1 for the striped skunk and 5:1 for mink, to 31:1 for polar bears and 52:1 for the Japanese bear [18, 20]. When it comes to monotremes and marsupials, the lactose in their milk (~18 mg/ml) is secreted in a form of tri- and tetra-saccharides or galactosyl oligosaccharides, respectively [13], with the majority of monotreme lactose bound to a fucosyl group [21]. Furthermore, marsupials alter the ratio of oligosaccharides to lactose in their milk across a lactation [22–24]. As an example, the milk of the tammar wallaby contains approximately 25 mg/ml lactose, which rises to around 39 mg/ml after 13 weeks. After 28 weeks, the lactose in their milk is cleaved within the mammary epithelium, thereby eliminating any free lactose from the milk for the remaining lactation. As a result of these changes the content of oligosaccharides and free glucose and galactose in the milk of the tammar wallaby increases as its free lactose content declines [25].

The synthesis of lactose by the mammary gland is also a major determinant of its milk volume output, where the concentration of lactose in milk is positively associated with its volume and negatively associated with the osmolarity of its salts [1]. A hypothetical model proposed in the 1970-80’s described the swelling of Golgi vesicles with water in response to lactose synthesis and accumulation, thereby offsetting the high osmotic potential of lactose as the consequence of its multiple hydroxyl groups [26]. Specifically, hydrogen bonds form between the hydroxyl groups of lactose and a molecule of water, whereas the ring oxygens and the bridging oxygen in lactose do not bind water [27]. The length of this hydrogen bond (fructose < sucrose < glucose < lactose ≪ mannose) in mono- and disaccharides is also negatively correlated with the sweetness of the carbohydrate [27]. Lactose has more opportunities for hydrogen binding and hydration than the inorganic salts in milk (i.e., Cl, Na), which explains why in most species the lactose concentration of milk is inversely correlated with its osmolality and positively correlated with milk volume. The inverse correlation between the concentration of lactose and inorganic salts in milk also maintains milk as isosmotic to blood [1], which is essential for sustained and optimal milk synthesis.

These unique biochemical properties of lactose highlight the important contribution that this seemingly simple carbohydrate makes to the nutritive value of milk, alongside its crucial role in the sustained transfer of essential water and solutes to the offspring. As a changing climate and reduced water availability threaten mammalian survival, there becomes a greater need to understand the critical role for lactose in milk across a range of species. However, given that laboratory mice and rats each produce different sets of oligosaccharides in their milk, as well as compared to human milk, the question must be raised as to which model organism is best-suited for studying lactose and oligosaccharide synthesis. In a related way, it is worth emphasizing that genetic selection for milk production in livestock has likely also shifted the relative abundance of lactose in milk, albeit the extent of such changes historically can be difficult to assess. The best evidence for such a shift comes from a long-range genetic selection study in Holstein dairy cows, where selection for production traits reflective of the modern industry saw cows have a higher lactose content in their milk, mostly during late lactation [28].

## Precursors, Genes, and Proteins Required for Lactose Synthesis

### Lactose Precursors and Mammary Hexoneogenesis

There is no doubt that lactose synthesis creates massive pressure on an animal’s metabolic balance, where the partitioning of maternal nutrients in support of lactation, coined “homeorhesis,” is critical to support ongoing milk synthesis [29]. To emphasize this point, the mammary glands of a dairy cow in peak lactation can use up to 85% of all circulating plasma glucose, where total glucose turnover can exceed 3 kg/d. Of the glucose that is taken up by the udder, between 65 and 70% is used to synthesize lactose [29]. Ultimately it is the mammary gland(s) that control glucose uptake and utilization from the circulation, although many questions remain as to precisely how they regulate the uptake of glucose in support of lactose synthesis [30, 31]. The effect of plasma glucose levels on lactose synthesis is further explored in our companion review [32].

Precursors delivered to mammary epithelial cells (MEC) in support of lactose synthesis primarily originate from plasma glucose, where gluconeogenesis by the liver plays an important role in maintaining plasma glucose levels. However, we must emphasize that plasma glucose is not the sole precursor for lactose synthesis and that glycerol and galactose are alternative carbon sources for lactose synthesis. In fact, the contribution of plasma glucose to lactose synthesis during fed and fasting states is consistently in the order of 80 and 60%, respectively [33–41]. Dietary or infused glucose contributed to approximately 80% of the lactose synthesized by non-fasted lactating humans, but to only 62% of the lactose synthesized in the fasted state [36, 40]. In the fed state, ≥ 98% of the glucose in lactose came from the plasma, whereas only 68% of uridine galactose (UDP)-galactose originated from plasma glucose. After a 24 hour (h) fast, 72% of the glucose and 51% of the UDP-galactose in human milk were derived from plasma glucose [36, 42]. Similarly, approximately 70% of the lactose produced by both high and low producing goats was derived from plasma glucose [34], which was similar to the incorporation of plasma glucose into 59% of lactose carbon produced by sows [43].

**Fig. 2:**
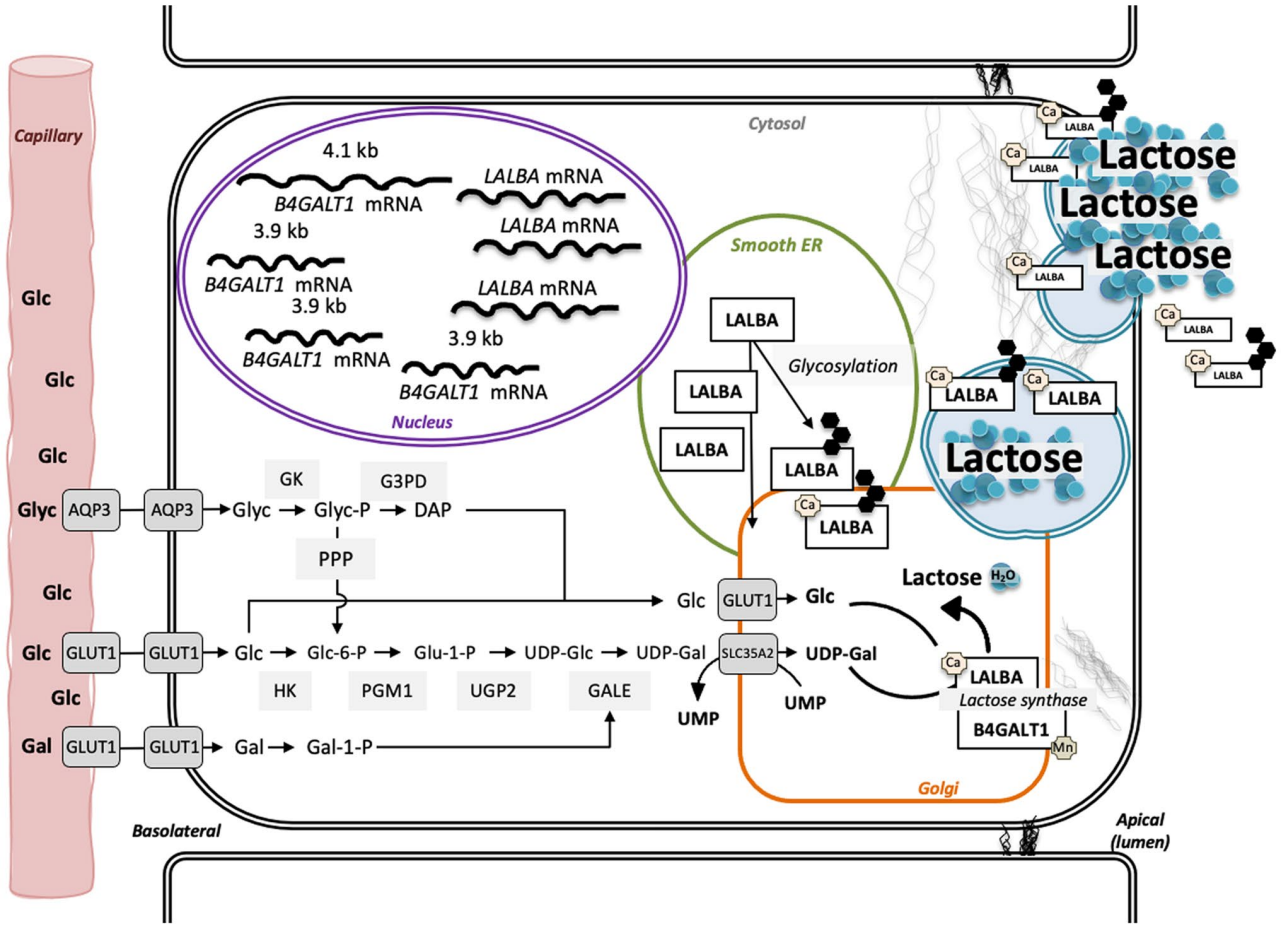
A schematic representation of the biochemical and cellular requirements for lactose synthesis. Glucose and non-glucose precursors are taken up by the mammary epithelial cell at its basolateral surface. Some glucose is shuttled directly to the Golgi while other glucose and non-glucose precursors are converted to UDP-galactose through a series of enzymatic reactions. The 3.9 kilobase B4GALT1 mRNA is preferentially and abundantly transcribed and translated during lactation relative to the 4.1 kilobase B4GALT1 mRNA. Some LALBA is glycosylated in the smooth endoplasmic reticulum. The lactose synthase complex is formed by B4GALT1 and LALBA in the Golgi, which then joins glucose and UDP-galactose to form lactose while the UMP moiety is recycled. Lactose, LALBA, and B4GALT1 within vesicles are secreted by exocytosis, and are guided and supported by microtubules and microfilaments. Abbreviations: Aquaporin 3 (AQP3), α-lactalbumin (LALBA), β-1,4-galactosyltransferase-1 gene (B4GALT1), calcium (Ca), dihydroxyacetone phosphate (DAP), endoplasmic reticulum (ER), galactose (Gal), glucose (Glc), glucose transporter 1 (GLUT1), glycerol (glyc), glycerol kinase (GK), glyceraldehyde-3-phosphate dehydrogenase (G3PD), hexokinase (HK), kilobases (kb), manganese (Mn), messenger ribonucleic acid (mRNA), pentose phosphate pathway (PPP), phosphoglucomutase (PGM), UDP-glucose-pyrophosphorylase 2 (UGP2), phosphate (P), solute carrier family 35 A2 (SLC35A2), uridine diphosphate (UDP), uridyl monophosphate (UDP), UDP-glucose 4-epimerase (GALE)

One alternative source of carbon for lactose synthesis is glycerol that can be taken up directly by MEC and converted to glucose and UDP-galactose *de novo.* Glycerol accounted for approximately 14–70% of the UDP-galactose synthesized de novo*,* and approximately 10% of newly-synthesized glucose in fed and fasted lactating humans [36, 40, 42]. Likewise, in fed and starved goats, 27% and 21% of the UDP-galactose moiety of lactose, respectively, was created *de novo* from glucose 6-phosphate, which resulted in the asymmetric labeling of its carbon [40, 44]. This process, coined mammary “hexoneogenesis,” generates hexose phosphates within MEC by integrating non-glucose precursors into the triose isomerase reaction or the pentose phosphate pathway [40, 45]. In the case of the triose isomerase reaction in MEC, glycerokinase phosphorylates glycerol, which is then converted to dihydroxyacetone phosphate by glycerol-3-phosphate-dehydrogenase. Dihydroxyacetone phosphate then feeds directly into the triose isomerase reaction within the glycolytic pathway (Fig. 2). A labeled precursor was more likely to have been recycled through the pentose phosphate pathway if the labeled glucose or UDP-galactose moieties within lactose had a higher C6-C4:C3-C1 ratio of enrichment [40, 44]. By contrast, when the glucose moiety in lactose is derived directly from plasma glucose, the distribution of its carbons is identical to that found in plasma glucose [37–41, 46].

In a similar way, galactose can also be taken up by MEC for its direct incorporation into lactose. Infusion of ^13^C-galactose into breastfeeding women yielded two ^13^C peaks in milk lactose, one within hours and the second a day later due to the incorporation of ^13^C-glucose derived from hepatic metabolism of ^13^C-galactose. Only the C1-atom of galactose and the C1-atom of glucose in lactose were labeled, suggesting that infused, labeled galactose contributed to both the glucose and UDP-galactose moieties [36, 42]. The direct incorporation of labeled plasma galactose into lactose indicates that MEC take up and route galactose to the Golgi, potentially through glucose transporter 1 (GLUT1) and UDP-galactose translocator (SLC35A2) [30, 36, 42].

These considerations regarding the supply and utilization of large quantities of substrate in support of lactose synthesis have broad implications for understanding the regulation and coordination of lactation physiology. Whereas the normal level of demand for glucose is already high, metabolic dysregulation during states ranging from obesity to undernutrition can quickly have a negative impact on lactation performance, while extreme states such as ketoacidosis can be fatal. Differential utilization of the various substrates during fed and fasted states highlights the need to further study how the endocrine environment regulates precursor mobilization as well as delivery to, and uptake by, the MEC. In essence, there is the need to refine our understanding of the factors controlling homeorhesis. As a step in this direction, we summarize some of the key hormones implicated in the regulation of lactose synthesis in our accompanying review [32].

### The Cell Biology of Lactose Synthesis

Lactose is produced exclusively in the Golgi apparatus of the MEC. Here we outline the pathway for lactose synthesis, assuming that all its carbon derives from circulating glucose. Extracellular glucose is taken up by MEC via GLUT1 and sodium-glucose transporter 1 (SGLT1), then transported into the Golgi apparatus via GLUT1 (Fig. 2) [30]. Glucose is then phosphorylated by hexokinase (HK) to yield glucose-6-phosphate, which is then used to create a pool of UDP-bound galactose in the cytoplasm. Several sequential steps then facilitate the *de novo* synthesis of UDP-galactose. First, phosphoglucomutase (PGM1-3) transfers a phosphate group from the C6 position of glucose-6-phosphate to the C1 position of glucose. Next, UDP-glucose-pyrophosphorylase (UGP2) exchanges the phosphate group for a UDP moiety. The resulting UDP-glucose is then converted to UDP-galactose by galactose epimerase (GALE). Alternatively, glucose-1-phosphate can first be converted to galactose via galactose epimerase (GALE), followed by the transfer of UDP to galactose-1-phosphate by galactose-1-uridyltransferase (GALT). UDP-galactose is then shuttled into the Golgi via SLC35A2 or SLC35B1 [47–49]. The final step of lactose synthesis occurs within the Golgi apparatus, where lactose synthase (LS) joins glucose and UDP-galactose by a β-1-4 glycosidic bond [50–52]. Importantly, LS is a unique enzyme complex comprised of β-1,4-galactosyltransferase-1 (B4GALT1) and the mammary-specific modifier protein α-lactalbumin (LALBA), and requires close association with the uridine nucleotide cycle on the trans-Golgi [53]. Once lactose is produced by LS, it is then packaged into vesicles from the trans*-*Golgi and transported to the apical membrane for exocytosis. With an eye to defining the control points for lactose synthesis, we characterize the individual proteins of the LS in the following sections, as well as the factors that regulate their interaction and activity.

### The Ubiquitous Enzyme, B4GALT1

**Fig. 3:**
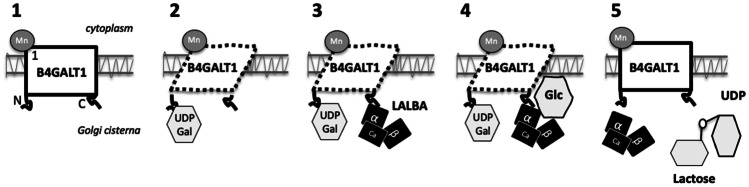
A graphical representation of the biomolecular process of lactose synthesis. (1) First, B4GALT1 resides in the Golgi in its inactive conformation. (2) Then, UDP-gal binds the N-terminus of B4GALT1. The enzyme shifts its conformation from an inactive to an active state, revealing the LALBA binding site. (3) Next, LALBA can bind B4GALT1, increasing the preference of B4GALT1 for glucose by 1000-fold. (4) Lactose synthase transfers D-galactose (derived from UDP-gal) to the OH-4 position of glucose to create lactose. (5) Lactose and LALBA dissociate and B4GALT1 returns to its inactive conformation. Abbreviations: α-lactalbumin (*LALBA*), β-1,4-galactosyltransferase-1 gene (*B4GALT1),* galactose (Gal), glucose (Flc), uridine diphosphate (UDP)

β-1,4-galactosyltransferases belong to a family of seven transmembrane proteins that are present in most secretory cells in the body. In the absence of LALBA, these Mn-dependent enzymes transfer D-galactose from UDP-galactose to N-acetylglucosamine [54]. Central to LS activity is B4GALT1, whose structure, function, and orthology across species has been reviewed extensively [54–56]. The B4GALT1 protein (Fig. 3) has two metal ion binding sites (sites 1 and 2), an N-terminal domain that recognizes the nucleotide donor (UDP-galactose), a C-terminal domain that recognizes the glucose acceptor, and an active site located between the two domains. The amino-terminus of the mature B4GALT1 protein is embedded within the Golgi membrane and requires that Mn be bound to site 1 (Fig. 3) for maximal activity [57]. This binding of Mn is an absolute requirement for the binding of UDP-galactose [58]. Site 2 is a low-affinity site that binds Mn or Ca, which serves the primary role of enhancing the efficiency of catalysis and the binding of glucose.

While the binding of Mn to site 1 of B4GALT1 is required for lactose synthesis to proceed (Fig. 3), high concentrations of MnCl are inhibitory [57, 59]. The activity of B4GALT1 was submaximal when ions such as Zn, Fe, Co were bound at site 1, while other ions including Na, K, Mg, and Ca did not affect B4GALT1 activity, as they could not bind site 1 [60]. It is the occupation of site 2 that determines the affinity of B4GALT1 for glucose [58]. The binding of Ca, Mg, ciopene, or spermidine to site 2 modified the ability of the enzyme system to synthesize lactose, emphasizing the complex role of B4GALT1 as a multi-ligand allosteric enzyme. Depending on which cation was bound to site 2, the K_m_ for Mn at site 1 could be lowered and the V_max_ raised, leading to the stabilization of glucose binding to LS [58].

### The Unique Modifier Protein, LALBA

Central to the activity and function of LS is LALBA, a protein that is expressed exclusively by MEC, and whose role has been reviewed extensively by others [61–63]. We should emphasize that beyond this role, LALBA secreted into milk is also important for infant nutrition due to its high tryptophan, lysine, and branched chain amino acid content [64]. In fact, human milk has one of the highest concentrations of LALBA (~ 2.4 mg/ml) which comprises more than a quarter of all its protein [64–66]. Perhaps not surprisingly, several lines of evidence also indicate that bioactive peptides derived from LALBA support maturation of the infant gut [64].

### Variation in LALBA Distribution

While we provide a more detailed overview of the transcriptional regulation of LALBA gene expression in the accompanying review [32], it is worth highlighting here that from a physiological context, the expression and distribution of LALBA within the mammary gland(s) is not as homogenous as might be implied in the general literature. Indeed, others have highlighted that heterogeneity in the expression of LALBA mRNA and protein in the sheep and goat udder can misrepresent the true level of LALBA gene activation and protein synthesis [67, 68]. *In situ* hybridization on biopsies from the udder of a 14 day prepartum ewe revealed that *LALBA* mRNA was expressed heterogeneously in isolated single MEC, in clusters, and in some lobules [69]. Likewise, MEC within collapsed alveoli contained few to no milk fat globules, but expressed high levels of *LALBA* mRNA, whereas MEC that contained abundant milk fat globules did not express *LALBA* mRNA. The *LALBA* mRNA transcript was also rarely detected in multilayered ducts such as in the gland cistern [69]. Similar transcriptional heterogeneity was also observed in the mammary glands of mice, where *LALBA* mRNA expression was uniform across the alveoli unless they were distended and the epithelium was flattened, or after the teats were sealed and milk stasis ensued. Notably, the expression of whey acidic protein (WAP) or β-casein mRNAs was not as heterogeneous [70], further highlighting that LALBA and other milk proteins are not expressed in perfect unison. As another demonstration of this heterogeneity, a comparison of total RNA from milk fat to that from biopsied mammary tissue or shed epithelial cells revealed that the abundance of LALBA transcripts was greater in the former [71]. In a similar way, Carli *et al.* recently identified that among MEC shed into human milk there were many functionally-distinct cells (> 35%) that they proposed might sub-specialize in lactose production, while others (4%) were suggested as being primarily responsible for the synthesis of milk proteins and lipids [72]. We suggest it is more likely that this differential expression of lactose, protein and lipid synthesis across MEC reflects the heterogeneity and acute temporal regulation of milk synthesis in the gland at any given time. That said, a long list of knowledge gaps remains regarding factors that might regulate the transcription and translation of LALBA across individual MEC, whether that be cell stretch, local feedback, or local blood flow, to name but a few. Regardless, we should stress that an appreciation for the heterogeneity of LALBA expression requires careful consideration when studying the physiological and local factors that regulate lactose synthesis.

### The Structure and Function of the LALBA Protein

Mature LALBA, which functions as an enzyme modifier, is a glycosylated 123 amino acid metalloprotein with a molecular size of ~ 14.5–18.5 kilodaltons. Its structure consists of a large α domain and a small β domain with a deep middle cleft that binds Ca [73, 74]. In keeping with the essential role of LALBA across species, parts of its sequence are highly conserved; specifically, there are thirty invariant amino acid residues in LALBA across all mammals, most of which are involved with binding B4GALT1 and Ca [21, 73]. A more detailed description of the role for various amino acids in the form and function of LALBA has been offered elsewhere [75, 76].

The LALBA molecule has several critical regions. Saccharide binding is achieved through the cleft region of LALBA, which is homologous to that in C-type lysozymes. The aromatic cluster I (AC1) within LALBA, specifically Leu-110, facilitates its interaction with B4GALT1 and is critical for LS activity [73, 74, 77]. The crucial nature of this AC1 site is highlighted by the fact that mutation of the AC1 flexible loop region (as occurs in otariids like the fur seal) or deletion (as occurs in the walrus) suppresses LALBA synthesis and leads to a milk devoid of lactose [10, 11]. The least flexible region of LALBA is the Ca-binding loop and a hydrophobic region, where residues Lys-79, Asp-82, Asp-84, Asp-87, and Asp-88, along with two molecules of water, support Ca binding [73, 77]. This Ca-binding loop is formed by one disulfide bond between residues 73 and 91, with additional stability provided by residues 61 and 77 [73, 77]. The number of Ca binding sites on LALBA varies by species as described elsewhere [74, 78]. Likewise, a full review of metal ion binding to LALBA has been provided by others [76, 78, 79]

### Post-translational Modification of LALBA

The LALBA protein is often glycosylated, yet the biological significance of this post-translational modification is poorly understood (Fig. 2). Importantly, both glycosylated and non-glycosylated forms of LALBA are active in LS and are secreted into the microsomal fraction of milk (Fig. 2) [80]. While there is abundant evidence in other protein and cell systems that N-glycosylation affects protein localization, stability, folding, and solubility, it remains unclear why LALBA is variably glycosylated [81]. Approximately 10% of bovine and murine LALBA is glycosylated, while only 1% of human LALBA is glycosylated [82–85]. In cats, the glycosylated and non-glycosylated forms of LALBA occur in equal ratios, whereas in rabbits LALBA is predominantly glycosylated [86, 87]. Rat LALBA is unique in that it is present in milk as three charged forms, each of which is glycosylated [88]. Compared to bovine LALBA, rat LALBA has four amino acid substitutions (Lys/Glu at 43, Asp/Asn at 44, Gly/Gln at 46, and Glu/Asp at 49), which likely facilitates its increased glycosylation. Moreover, the secondary structure of the peptide sequence required for N-glycosylation in rat LALBA was predicted to have a ß-bent conformation, which would further enable B4GALT1 to access the glycosylation site [88]. The site of glycosylation in human LALBA is disputed, as glycosylation has been variably detected at the Asn45 site and at amino acid 71 (Asn-71-Ile-Cys), which is an amino acid triplet that is conserved in all LALBA except that of the red-necked wallaby [83, 89, 90]. Interestingly, when human LALBA was overexpressed in the udder of dairy cows, the transgene product was not glycosylated, whereas the endogenous bovine LALBA became unusually glycosylated at Asn-71 [91]. How these forms of LALBA differentially modulate lactose synthesis, or how the extent of glycosylation is regulated, still remains unclear.

### Factors Regulating the Interaction between LALBA and B4GALT1

The interaction between LALBA and B4GALT1, which occurs in the Golgi apparatus in a specific order, is critical for LS activity and involves specific metal ions (Fig. 3). Importantly, we should point out that *in vitro* studies of metal ion binding to LALBA were performed in the absence of B4GALT1, leaving it unclear as to whether the multiple conformations of LALBA described in vitro also occur *in vivo*, along with questions regarding the relevance of these to lactose synthesis. Only B4GALT1 interacts with UDP-gal (Fig. 3). Once UDP-gal binds the N-terminus of B4GALT1, the enzyme shifts its conformation from an inactive to active state, revealing the LALBA binding site [55, 74, 92, 93]. Fascinatingly, this binding of LALBA to B4GALT1 subsequently increases the preference of B4GALT1 for glucose by 1000-fold [55, 74], where hydrogen bonding maintains glucose in the catalytic site. Within the resulting LS complex, subsite F is positioned close to the galactosyl acceptor subsite of B4GALT1 to establish favorable interactions for glucose. What remains unclear is how the interactions of LALBA with glucose are stabilized by B4GALT1, where it has been suggested that AC1 residues participate in stabilization together with subsite F [77, 94]. Because only a monosaccharide-sized binding site becomes available, extended sugars like N-acetylglucosamine cannot bind B4GALT1 in the presence of LALBA [55, 74, 92]. The LS then transfers D-galactose (derived from UDP-galactose) to the OH-4 position of glucose to create lactose (Fig. 3), after which lactose and LALBA dissociate and B4GALT1 returns to its inactive conformation [55, 74, 92].

### The Regulation of LS Activity

There are a number of factors that regulate and specify LS activity. To start with, there likely is a degree of functional complementarity between LALBA and B4GALT1 for a given species. As an example, LALBA isolated from the platypus did not facilitate lactose synthesis when paired with bovine B4GALT1, where the concentration of platypus LALBA required for optimal lactose synthesis was 20-fold higher when it was paired with bovine B4GALT1 [21]. In addition, disruptions in the acid–base balance and the concentration of ions can inhibit LS activity. For example, hydrogen protons are a byproduct of glycosylation, where the Ca^2+/^Mn^2+^ ATPase 1, TMEM165, works as a hydrogen exchanger to deacidify the Golgi. In keeping with this critical function for TMEM165, its conditional deletion in the mammary glands decreased LS activity and milk lactose content by 36% [95]. Likewise, a high concentration of K inhibited LS activity *in vitro*, but only in the concentration range at which potassium was bound to LALBA [79].

The provision of various non-glucose precursors, analogs, or intermediates can also interfere with and/or inhibit LS activity. For example, glucose analogs such as 4-deoxy-D-xylo-hexose and 4-azido-4-deoxy-D-glucose suppressed the ability of LALBA to bind glucose, while N-acetylglucosamine acted as non-competitive inhibitor by changing the conformation of B4GALT1 from an inactive to an active state [96], thereby preventing the binding of LALBA or UDP-galactose due to steric hindrance. Likewise, the presence of LALBA at extremely high concentrations leads to it binding B4GALT1 while it is still in its active state, creating a complex comprised of B4GALT1, UDP-galactose, Mn, and LALBA that then precludes glucose binding due to steric inhibition [74]. Finally, uridine triphosphate also inhibited LS activity, although the mechanism is not understood [59]. Combined, despite the massive amount of LS activity and turnover within MEC, there clearly is a parallel sensitivity to the biochemical microenvironment that has barely been unearthed at the physiological and molecular level, especially when considering the heterogeneity of LALBA expression within the gland we mentioned earlier.

### Secretion of Lactose

Once synthesized, large quantities of lactose are rapidly packaged into secretory vesicles (Fig. 2**)** alongside other proteins and ions for export from MEC by exocytosis. While it is well-established that vesicles containing milk proteins, lactose, and water are formed in the trans*-*Golgi, the way in which fragile and osmotically-active secretory vesicles are transported to the apical membrane of the MEC is not well-defined and warrants further investigation [97–99]. Generally speaking, MEC utilize a combination of microtubules and microfilaments to direct secretory vesicles from one organelle to another and toward the apical membrane [100, 101]. In keeping with this mechanism, agents that inhibited microtubule function disrupted milk secretion in goats, rats, and guinea pigs also decreased lactose secretion as well as the accumulation of glucose, pyruvate, citrate, glycerol, and lactate in MEC [100, 102–105]. Similar outcomes, including a 50% reduction in glucose uptake and a 50% reduction in lactose content and LS activity occurred when microfilaments were inhibited in mammary explants from lactating guinea pigs [101, 104]. Once secreted, LALBA can also dimerize with itself, creating a potential feedback inhibitor that can promote MEC apoptosis via inhibition of histone deacetylase activity [106]. Without doubt, there is a great deal that remains to be learned about secretory vesicles in MEC, their transit, and the expulsion of their contents into the alveolar lumen.

## The Physiology and Timeline of Lactose Synthesis

Having defined the biochemical pathway of lactose synthesis, our next objective is to review the temporal changes that occur physiologically as part of the upregulation and maintenance of lactose synthesis during lactation. Not only does this consideration provide further insight into the regulation of lactose synthesis, but it also affords important information that can improve the translation relevance of this pathway.

### The Onset of Lactose Synthesis During Pregnancy and Lactation

Lactose synthesis by MEC can be first detected in mid- to late gestation during a period of secretory differentiation (also known as lactogenesis I), which corresponds to an increase in the size and number of organelles in MEC, and a small but appreciable increase in milk protein gene expression [107, 108]. During this period the secretions that have accumulated in the alveolar lumen can diffuse paracellularly between MEC into the blood prior to their excretion in urine [107], such that lactose can be detected in the urine of pregnant humans by the second trimester [109, 110]. Others reported that the concentration of LALBA (8 ng/ml) in the plasma of pregnant humans was stable between 28 and 14 weeks prepartum, and increased to a peak of greater than 1 μg/ml at parturition [111].

Lactose synthesis increases rapidly during the subsequent acute phase of secretory activation (also known as lactogenesis II). The timing of periparturient secretory activation is associated with a rapid decrease in circulating progesterone, albeit timing varies by species [108, 112]. Secretory activation in humans occurs postpartum as plasma progesterone levels decline rapidly following delivery of the placenta, whereas in other species such as pigs and rats, secretory activation occurs prepartum when plasma progesterone levels decrease [112, 113]. Secretory activation also coincides with the sealing of tight junctions at the apical border between MEC, concomitant with the dramatic increase in the transcription of genes in support of copious milk production [107, 114]. As discussed in our subsequent review [32], these and other changes within MEC are directed by a general increase in circulating glucocorticoid, insulin, and prolactin levels, and a decline in circulating progesterone and estrogen, where these changes are species-specific [108, 112].

The onset of lactose synthesis and secretory activation can be detected by a number of means. A clinical indicator in humans can be the self-report of breast/chest fullness, although a more sensitive and reliable biomarker is the rapid decrease in the Na/K ratio in milk [115, 116]. Likewise, the level of lactose and/or LALBA in milk, urine, or plasma are also excellent biochemical indicators of secretory activation. For example, a decrease in lactose and LALBA in plasma and urine paralleled a drop in circulating progesterone in humans [117, 118], while in cows, serum LALBA decreased to 140 ng/ml at L14 from a peak of 1000 ng/ml at parturition [111].

Much of the information regarding the onset of secretory activation was obtained by assaying LS activity in mammary tissue slices, either by measuring activity of the functional complex or the individual activity of LALBA or B4GALT1. In all these cases, enzyme activity was expressed as nmol of lactose produced per min per mg of particulate protein. Using this approach, LS activity in mammary tissue slices from cows increased by 1.4 units between L(-30) and 7 days prepartum L(-7), and by another 3 units by L(40). Concomitant with the increase in LS activity, LALBA concentration in bovine mammary tissue increased from undetectable levels at L(-30) to 82 and 178 μg/g per wet weight of tissue by L(-7) and L(7), respectively [119]. Likewise, activity of LS in mammary tissue of goats was detectable by day 120 of pregnancy (G120) even in the presence of high plasma progesterone levels [67, 68]. Similarly, in rodents, 20–30% of the rise in LS activity in mammary tissue occurred by G20 [120–122]. Prior to G16-18, mice had limited LS activity (1–3 ng/h/mg wet weight), which then increased to 33 ng/h/mg wet weight between G19 and 8 h postpartum. The activity of the LS was highest in lactating mice from L(2) through L(6) at 142 ng/h/mg wet weight [123].

In recent decades the study of lactose synthesis onset has shifted to the transcriptomic analysis of genes within the pathway. One challenge that precludes a clear definition of the genetic regulation of the timing of this onset and its rate-limiting factors has been the lack of temporal standardization for transcriptomic and proteomic analyses. Consistent with the aforementioned relationship between the sealing of tight junctions and secretory activation, Lemay *et al.* suggested that the best practice was to cluster gene expression signatures relative to the milk Na/K ratio [115]. In the following discussion of physiological processes, we include insights into the transcriptomic changes that occur in the mammary gland during secretory activation across species. We should point out that in the studies described below, differential gene expression was rarely correlated with changes in milk composition or volume.

### The Timing of Glucose Uptake Onset and Its Conversion to UDP-galactose

The uptake of glucose into the mammary epithelium is central to the initiation and maintenance of lactose synthesis. In dairy animals there is wide variation in the relative increase in GLUT1 gene and protein expression during secretory activation [47, 49, 124]. In humans, GLUT1 increased 1.4-fold between 6 h and L(7) in MEC, while GLUT1 and GLUT10 increased 7 and 8-fold, respectively, by L(4) from 6 h postpartum [48]. In mice and rats, GLUT1 gene expression increased approximately 3-fold by L(2) relative to that in late gestation [125, 126]. Even though these data confirm a well-established increase in GLUT1 gene and protein expression in MEC around secretory activation, there is no clear consensus as to the level of GLUT1 gene expression required for maximal lactose synthesis [30].

Interestingly, limited information also exists regarding the temporal expression of genes within the lactose synthesis pathway around the time of secretory activation, outside of for *GLUT1* and *LALBA*. In human milk fat globule membranes first collected 6 h postpartum and then every 12 h for, 4 days as a source of MEC-derived RNA, expression of *HK1, HK2*, and *HK3* mRNA was decreased, while that for *PGM1, GALK1, GALK2, PGM2, UGP2, GALE, GALT*, and *SLC35A2* was increased by L(4) compared to baseline samples. While the greatest fold-change in gene expression was recorded for *GALK2, UGP2*, and *PGM1–3* by L(4) (relative to 6 h postpartum), only the expression of *UGP2, PGM1*, and *SLC35A2* was correlated with milk lactose concentration [48]. Similarly, in another study of milk fat mRNA obtained from breastfeeding patients and stratified by milk Na/K ratio to define colostrum, transitional, or mature milk, the expression of *SLC2A9, GALK1, PGM1, UGP2, GALE,* and *SLC35A2* was increased in transitional milk compared to that in colostrum, whereas the expression of *GALT* and *HK1* was unaltered [115]. In contrast to the change in hexokinase expression recorded in human MEC, the expression of *HK1* in the MEC of sows increased after parturition and maintained that level throughout lactation, while *HK2* expression increased 2.5-fold within 12 h of parturition, then returned to levels recorded in pregnancy by the end of lactation [47]. The expression of *SLC35A2* increased 1.88-fold by L(14) and then decreased by L(21), while the protein expression of *SLC35A2* increased by L(4) and then plateaued [49]. In lactating rats, the *Hk1* gene was expressed in mammary tissue samples from both pregnant and lactating rats, whereas *Hk2* was expressed only during lactation after its expression increased 2.44-fold by L(1.5) [126, 127].

As highlighted in this and the previous section regarding mammary hexoneogenesis, lactose synthesis has a high degree of plasticity, allowing MEC to up- or down-regulate various biochemical pathways to ensure that milk synthesis is optimal at all times. The regulation of genes required for the conversion of glucose to UDP-galactose, such as PGM1 and UGP2, should be further examined, given that in humans the expression of PGM1 and UGP2 was strongly associated with changes in milk lactose concentration.

### Temporal Changes in B4GALT1 Gene and Protein Expression

There is no doubt that the expression of B4GALT1 is a parallel key driver for the onset of lactose synthesis. At the genetic level, mRNA for *B4GALT1* is expressed in most cell types across various tissues and has a long 5’ UTR with an extensive secondary structure. Importantly, to support lactation MEC increase their transcription of a 3.9 kb B4GALT1 mRNA variant that has a shortened 5’UTR and increased translational efficiency (Fig. 2). It is this 3.9 kb *B4GALT1* mRNA transcript that helps support the rapid increase in lactose production in the early postpartum period [128, 129]. That said, there is discordant evidence as to whether the expression of B4GALT1 in MEC is upregulated in the first week postpartum across species. Specifically, whereas the expression of the B4GALT1 gene was increased in RNA isolated from the milk fat globule membrane of colostrum samples compared to mature human milk in one study, it did not change over time in another [48, 115]. In the MEC of pigs, expression of *B4GALT1* increased 3.34-fold between L(-3) and L(0), then plateaued by L(2) [47, 49].

### Temporal Changes in LALBA Gene and Protein Expression

Not surprisingly, the expression of *LALBA* in the mammary gland increases dramatically during secretory activation so as to facilitate the rapid onset of lactose synthesis. Transcripts for *LALBA* mRNA were undetectable in pig mammary tissue prior to G(90) then increased 156-fold between L(-14) and L(-2) [46, 49, 129, 130]. In dairy cows, LALBA protein was not detected in mammary tissue at L(-30) [119], while in nulliparous sheep, *LALBA* mRNA was first detected in mammary tissue at L(-14) when its expression in MEC was heterogeneous [69]. Similarly, LALBA was first detected in mammary tissue of goats around mid-pregnancy [68]. In pigs, the level of *LALBA* mRNA in mammary tissue increased 11.8-fold between L(-14) and L(-3), and by another 1.3-fold between L(-3) and L(1) [47, 49]. Between L(-5) and L(10), the expression of *LALBA* in mammary tissue from cows did not change [124].

As mentioned earlier, there are various ways that LALBA levels can be monitored to track the onset of secretory activation. The level of LALBA in the plasma of pregnant humans varies between individuals and over the course of a pregnancy, with the average value being 35.4 ng/ml within a range of 0 to 600 ng/ml [132]. While LALBA excretion into human urine has not, to our knowledge, been measured, the excretion of lactose into urine begins to rise between weeks 10 and 20 of gestation [110]. The concentration of LALBA in serum in early gestating heifers did not exceed 5 ng/ml prior to day 160 prepartum, then rose to 23-30 ng/ml between days 120 and 60 prepartum. In dairy cows the concentration of LALBA in the plasma increased from 221 ng/ml on L(-4) to 919 ng/ml on L(0), then declined to plateau at 463 ng/ml by L(2) [133]. Plasma levels of LALBA in goats began to rise 10-12 weeks prepartum concurrent with proliferation of the alveolar epithelium [68]. In pigs the level of LALBA in the blood increased rapidly between L(-7) and L(-2), coincident with an increase in plasma prolactin and a decrease in plasma progesterone [130, 131]. The LALBA protein could not be detected in the serum of pregnant rats [134].

Once lactation is established in humans, dairy animals, and rodents, the expression of the LALBA gene and its protein becomes relatively constant. However, this assertion must be weighed against the previous discussion about the potential for considerable heterogeneity of LALBA expression within the lactating gland. The LALBA mRNA transcript was one of the most abundant in the milk fat globule membrane isolated from human milk on both L(0.5) and at L(42) [48], where the content of LALBA in human milk peaked at over 4.9 mg/ml in the first few days postpartum, then decreased to 3.4 mg/ml a month later [135]. At the global level, the concentration of LALBA in milk from lactating humans in the United States was higher than from those in eight other countries (~ 3.4 mg/ml versus 2.4 mg/ml). In Mexico, for example, the average concentration of LALBA in human milk was only 2.1 mg/ml [65]. Bovine milk contains 1.2–1.5 mg/ml LALBA, which represents approximately 50% of all the whey proteins [136]. Equine milk is more similar to human milk, having a LALBA concentration of 2.4 mg/ml that represents ~ 30% of all whey proteins [136]. There is also substantial variation in the content of LALBA in the milk from mares, which ranges from 0.63 mg/ml to 2.94 mg/ml, depending on the breed and study [137]. Murine milk contains only 0.9 mg/ml of LALBA [138], while in the milk of rats, LALBA content varied between 1.5 and 8.5 mg/ml depending on stage of lactation [139]. In the Tammar wallaby, the LALBA content of milk (2.1 mg/ml) remained constant over a 40-wk lactation, even though the lactose concentration varied widely due to the progressive increase in its degradation into glucose and galactose as lactation progressed [25].

## Conclusion

The synthesis of lactose plays a critical role in directing the optimal growth and development of the young across nearly all mammalian species. At the broadest level, milk lactose content regulates milk osmolarity and overall milk volume and has important implications for water utilization across a drying planet. Lactose is also the building block for complex tri- and oligosaccharides that, until recently, could not be precisely analyzed. There is also a rising appreciation for the importance of lactose in human milk volume regulation, neonatal nutrition, and immune system development.

While the general biochemistry of lactose synthesis was elegantly defined within the last 50 years or so, a range of processes remain to be elucidated. The interaction between LALBA and B4GALT1 has been primarily studied *in vitro,* yet many questions remain as to how changes in the level of intracellular metabolites, including glucose, affect LS activity *in vivo*, whether that be in humans or dairy animals. Likewise, the function and regulation of the glycosylation of LALBA has yet to be determined. Similarly, the extrinsic and intrinsic factors that regulate *PGM, UGP2, GALE, GALT, SLC35A2* expression should be defined as these genes appear to be rate-limiting for lactose synthesis during secretory activation.

Clearly, there is also an ongoing need to better understand the regulatory strategies that fine-tune the synthesis of lactose at the level of the whole animal, the mammary gland, and MEC, which also varies across species. Even though plasma glucose is the main precursor for lactose, non-glucose precursors contribute up to 40% of carbon required to form lactose and have been largely overlooked. The role and regulation of these precursors warrants further investigation, particularly in the context of metabolic syndromes involving chronic inflammation and disrupted homeorhesis, such as ketosis, obesity, and diabetes mellitus. At the same time, questions remain as to how the regulation of lactose synthesis affects the production of tri- and oligosaccharides. We continue this theme of highlighting various extrinsic and intrinsic sources of regulation within the second review [32], where we present a range of opportunities to modulate milk composition through the regulation of lactose synthesis.

The cross-species diversity in lactose synthesis also underscores the importance of selecting the appropriate *in vivo and/or ex vivo* model for studying lactose production and its regulation. There is an ongoing need to develop a *bona fide in vivo or ex vivo* system to define the effect of ions, glucose, UDP-galactose, lactose, pH, and hormones on LS activity. The field still also needs an authentic model of lactose secretion so as to allow the closer study of the mechanisms by which lactose is packaged, transported, and exported from MEC into milk. Taken together, a great deal remains to be understood about lactose, a molecule that is all too often mistaken for being just a small and simple component of milk.

## Data Availability

Data will be made available by the corresponding author upon request.

## Abbreviations

LALBA: α-Lactalbumin
CSN2: β-Casein
B4GALT1: β-1,4-Galactosyltransferase-1 gene
L#: Day of lactation
L(-#): Day prepartum
GALE: Galactose epimerase
GALT: Galactose-1-uridyltransferase
G: Gestational day
GLUT: Glucose transporter
HK: Hexokinase
h: Hour
INS: Insulin
LII: Lactogenesis 2
LS: Lactose synthase
MEC: Mammary epithelial cell
min: Minute
PGMI-3: Phosphoglucomutase
SGLT1: Sodium-glucose transporter 1
SLC35A2: UDP-galactose translocator
UGP2: UDP-glucose-pyrophosphorylase
UDP: Uridine diphosphate.
